# Dielectric Properties of Water with a Low Quantity of NaCl inside Charged Nanoslits

**DOI:** 10.1021/acs.jpcb.4c06051

**Published:** 2025-02-04

**Authors:** Raúl Fuentes-Azcatl

**Affiliations:** Instituto de Física “Luis Rivera Terrazas’, Benemérita Universidad Autńoma de Puebla, Apdo. Postal J-48, Puebla 72570, Mexico

## Abstract

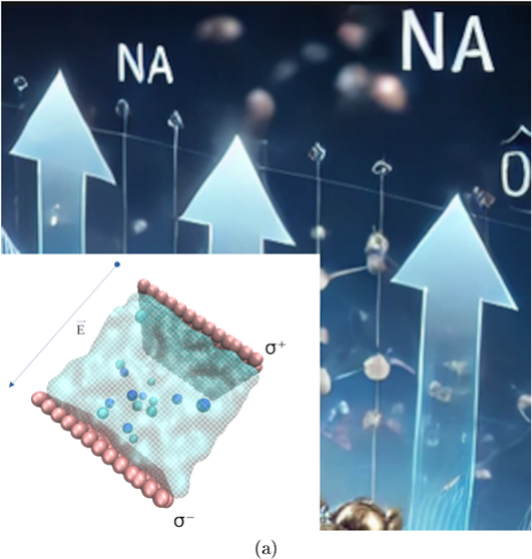

A 0.5 molal solution of NaCl in water confined within charged graphene nanoslits represents an intriguing system for molecular dynamics simulation, functioning as a model for a nanocapacitor. This charged configuration not only holds practical significance for the advancement of nanoscale capacitors but also offers valuable insights into how the charged walls and applied electric field influence the structure of water, the movement of ions within the solution, and how these alterations in water impact the overall fluid behavior. The behavior of the solution under nanoconfinement diverges markedly from that observed in bulk conditions, exhibiting distinct structural, dynamic, and dielectric properties. The charging of the graphene nanoslits generates an electric field within the nanopore, which plays a critical role in modulating molecular interactions. Key properties, including the static dielectric constant, polarization, and density of the 0.5 molal solution, are systematically examined through the molecular structure of the confined system. The models employed in this study include the flexible FAB/ϵ model of water, which effectively reproduces various experimental properties of water under different pressure and temperature conditions. Additionally, the NaCl/ϵ model is used, which also captures a range of experimental characteristics associated with sodium chloride solutions. Together, these models facilitate a comprehensive understanding of the complex behavior of water and ions under the influence of nanoconfinement and electric fields, providing insights that are essential for both fundamental science and practical applications in nanotechnology.

## Introduction

The extensive adoption of mobile devices like cell phones has led to the proliferation of rechargeable technologies such as lithium-ion batteries and supercapacitors, driven by significant advancements in materials science.^1^ Progress in developing new materials for electrodes, dielectrics, and electrolytic systems has enabled engineers to refine the parameters of charge accumulators.^2,3^ This dynamic field of research is thriving, especially with the advent of sodium batteries.^4^ Research teams globally are investigating various combinations of electrodes, solvents, cations, and anions to achieve faster charging times, reduced degradation, and enhanced capacities. Understanding the behavior of ions in the electrolytic solution, both in the liquid phase and near the electrode surface during the charged state, is crucial for creating high-performance energy sources. It is crucial to understand and seek new materials to enhance the efficiency of these systems. Knowing that storage capacity is determined by the energy difference between the charged state (ions adsorbed on the electrode) and the discharged state (solvated ions) is fundamental.^5−7^

Porous materials have garnered considerable interest across numerous research fields due to their advantageous structural properties. Specifically, meticulously engineered mesoporous structures, featuring two- or three-dimensionally interconnected pores, have been identified as exceptionally promising electrode materials for high-performance electrochemical capacitors (ECs or supercapacitors).^8^ These structures provide a large surface area and facilitate efficient ion transport, which are crucial for enhancing the energy storage capabilities and overall performance of ECs. Consequently, ongoing research is dedicated to developing and optimizing these mesoporous materials to further improve the efficiency and effectiveness of electrochemical capacitors, recent progress in the design of mesoporous electrode materials for ECs, from electric double-layer capacitors (EDLCs)^9,10^ and pseudocapacitors (PCs)^11^ to hybrid supercapacitors (HSCs).^12^ These supercapacitors outperform secondary batteries in terms of durability and energy output. They typically rely on nanoscale porous carbonaceous materials, and their operation does not involve chemical reactions between the electrolyte and the electrode. Among the most widely used electrolytes in supercapacitors is an aqueous solution of sodium sulfate. Moreover, nanoscale carbonaceous materials have shown exceptional performance, providing multiple pores whose sizes match those of charge carriers. Graphite, graphene, activated carbon, and versatile carbon nanotubes are exemplary materials for the cathode and anode of supercapacitors. Emerging electrode materials, such as graphene-based composites, increase capacity and improve battery performance at lower temperatures, primarily due to their high surface area and thermal conductivity. Documenting the interactions between alkali ions and graphene is essential for advancing lithium-ion battery technology.

Molecular dynamics is a powerful tool for studying various systems. In this context, we investigate a mixture of NaCl in water at a concentration of 0.5 molal as a dielectric within two parallel graphene nanoslit structures. This research is part of a broader initiative aimed at understanding the forces governing systems confined in graphene nanoslits with surface charges that generate an electric field.^13,14^ The goal is to obtain data under different scenarios and propose new dielectrics for supercapacitors.

Our research focuses on the dielectric and structural properties of alkali metal cations, specifically sodium, in water confined between two graphene nanoslits in charged and discharged states, ranging from 0 to 5 V, which is the acceptable limit without causing graphene to melt.^14,15^ To maintain system neutrality in the simulations, we use graphene nanoslits with the same number of atoms but different charges, generating a potential difference and, consequently, an electric field. By simulating a charging process, we observe changes in the subelectrode region in response to the electrostatically driven adsorption of ions. We have elucidated the effects of the explicitly treated solvent during charging and in the neutral state.^16^

In this work, we employ the epsilon force field for both NaCl/ϵ^17^ and water with the FBA/ϵ model;^18^ where the NaCl/ϵ force field enhances the reproduction of the properties of the salt in solid and liquid phases and, furthermore, when combined with water, reproduces the properties of the mixture.

Our paper is organized as follows. In section “Model and Simulation Details” we introduce the water and nanopore models and the details about the simulation method. In section “Results” we show and discuss our results, and the conclusions are presented in section “Conclusions”.

## Model and Simulation Details

### Water Model

In this study, we utilized the recently proposed FAB/ϵ water model,^18^ which incorporates flexibility to facilitate the examination of structural changes induced by other substances, such as ionic liquids.^19^ This model is fully flexible, featuring harmonic potentials at the O–H bond and the angle formed by the three atoms, this model is part of a family of water models that have improved the reproduction of various properties of water.^20−22^ This flexibility enhances the force field’s accuracy, as demonstrated in previous studies involving the CO_2_ molecule.^23^ By employing this model, we aim to better understand the interactions and structural dynamics in systems involving various substances, thereby improving the predictive capabilities and application potential of the force field.
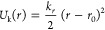
1and in the H–O–H angle,
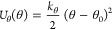
2where *r* is the bond distance and θ is the bond angle. The subscript 0 denotes their equilibrium values, and *k*_*r*_ and *k*_θ_ are the corresponding spring constants.

For the intermolecular potential between two molecules the LJ and Coulomb interactions are used for nonpolarizable models,
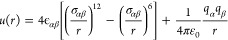
3where *r* is the distance between sites α and β, *q*_α_ is the electric charge of site α, ε_0_ is the permittivity of vacuum, ϵ_α_ β is the LJ energy scale and σ_α_ β the repulsive diameter for an α–β pair. The cross interactions between unlike atoms are obtained using the Lorentz–Berthelot mixing rules,
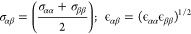
4

The Table 1 shows the force field values of the model mentioned above.

**Table 1:** Parameters of the Three-Site Water Model Considered in This Work

model	*k*_b_ (kJ/mol Å^2^)	*r*_OH_ (Å)	*k*_a_ (kJ/mol rad^2^)	Θ (deg)	ε_OO_ (kJ/mol)	σ_OO_ (Å)	*q*_O_ (e)	*q*_H_ (e)
FBA/ϵ^18^	3000	1.027	383	114.70	0.792324	3.1776	–0.8450	0.4225

### Graphene Model

The graphene walls in our model were represented as highly oriented pyrolytic graphite (HOPG) sheets, which are known for their high purity and well-ordered synthetic graphite structure. HOPG is characterized by a low mosaic spread angle, signifying that the individual graphite crystallites are closely aligned with one another. The initial force field for the carbon atoms in this graphene model is primarily defined by a Lennard-Jones interaction^26^ (refer to Table 2).

**Table 2:** Force Field Parameters of Graphene Model

model	σ/Å	(ϵ/*k*_B_)/K
C	3.401	35.46

This approach allows for a detailed simulation of the interactions between the graphene sheets and other molecules within the system. The choice of HOPG is particularly advantageous due to its well-documented physical properties and its ability to closely mimic the behavior of real graphene. By employing the Lennard-Jones potential, we ensure accurate modeling of the van der Waals forces between carbon atoms, which are crucial for understanding the material’s mechanical and thermal properties. This setup is essential for investigating various phenomena, such as adsorption, ion transport, and electrochemical performance, within the graphene nanoslits.

Charges are included in every carbon atom to maintain uniform surface charge densities, σ_*S*_, as given by Shim et al.^33^ The charges are increased from zero (neutral confinement) to create the desired voltage between the walls (see Table 3).

**Table 3:** Surface Electric Charge Density and Voltage Used in This Work^a^

q_*C*_ (e)	Q_graphene_ (C)	area (m^2^)	σ = Q/A (C/m^2^)
0	0	9.63 × 10^–18^	0
1.39 × 10^–04^	8.09 × 10^–21^	9.63 × 10^–18^	8.41 × 10^–04^
2.78 × 10^–04^	1.62 × 10^–20^	9.63 × 10^–18^	1.68 × 10^–03^
4.16 × 10^–04^	2.43 × 10^–20^	9.63 × 10^–18^	2.52 × 10^–03^
5.09 × 10^–04^	2.97 × 10^–20^	9.63 × 10^–18^	3.08 × 10^–03^

a Each nanoslit has 364 carbon atoms.

### NaCl Model

A limitation of molecular dynamics is that force fields do not always accurately reproduce the diversity of experimental data, which can lead to erroneous conclusions. The NaCl/ϵ^17^ model improves the reproduction of various properties in both liquid and solid phases. Additionally, when mixed with water, it enhances the reproduction of various thermodynamic properties of the mixture at different concentrations. Consequently, our research group has made significant strides in developing better force fields, which have been adapted to other models with minimal changes,^34^ corroborating our efforts to find improved force fields.

The parameters for the NaCl/ϵ model were fitted to match the experimental density of the NaCl crystal at a temperature of *T* = 298 K and a pressure of 1 atm, specifically, 2.16 g cm^–3^. This accuracy extends to various temperatures and also reproduces surface tension effectively, Table 4.

**Table 4:** Force Field Parameter of NaCl/ϵ

model	q/e	σ/Å	(ϵ/k_B_)/K
Na	0.885	2.52	17.44
Cl	–0.885	3.85	192.45

## Simulation Details

In our quest to comprehensively understand the intricate dynamics of confined of [NaCl] and [H_2_O] at low concentration, Molecular Dynamics (MD) simulations within the NVT ensemble were meticulously employed. The focal point of our investigation is a carefully designed nanocapacitor, crafted with precision using two parallel sheets of single-layer graphene (SLG). Each SLG sheet, meticulously arranged to accommodate 364 carbon atoms, spans an area defined by dimensions L_*x*_ = 3.1 nm and L_*y*_ = 3.1 nm. These graphene sheets, serving as confinement boundaries, are separated by a precise distance of L_*z*_ = 3.7 nm, thus creating a well-defined spatial enclosure for our confined [NaCl] and [H_2_O] system, where the density in the bulk is 1018 kg/m^3^.

Through these intricately designed simulations, we aim to unravel the subtle nuances of molecular interactions and structural transformations inherent within nanoscale confinement environments. By exploring the behavior of both [NaCl] and [H_2_O] within this nanocapacitor framework, our study promises to offer profound insights into the fundamental physicochemical phenomena governing the behavior of ions and water molecules in confined settings. Such insights hold immense potential for informing the design and optimization of nanostructured materials and devices for a myriad of technological applications.

Initially, the solution is 0.5 molal with a total of 848 water (H_2_O) molecules, along with 16 [NaCl] ions—comprising 8 Na and 8 Cl ions–were randomly distributed within the confined space of the nanocapacitor. Subsequent simulations were conducted with surface charge densities ranging from 0 to 40 V.

The establishment of an electrical potential within the system was achieved by introducing point charges onto each carbon atom of the single-layer graphene (SLG) parallel plates. This process facilitated the attainment of desired surface charge densities, as delineated in Table 3. Notably, the left wall served as the positive plate, while the right wall functioned as the negative one, ensuring a net charge of zero across the system.

All simulations were conducted using the molecular dynamics package GROMACS 2020.^35^ Periodic boundary conditions were imposed in the *xy*-plane. The equations of motion were solved using the leapfrog algorithm^35,36^ with a time step of 1 fs. The temperature was set to *T* = 298 K with the Nos’e-Hoover thermostat^37^ and a coupling parameter of 0.6 ps. The LINCS algorithm was employed for rigid bonds. The Lennard-Jones potential was truncated at 10 Å without any long-range correction in energy or pressure. The real part of the Coulomb potential was also truncated at 10 Å. The Fourier component of the Ewald sums was evaluated with the particle mesh Ewald (PME) method^38^ with a grid spacing of 0.35 Å and a three-degree polynomial for the interpolation.

All simulations were run for 50 ns, with the first 5 ns used as equilibration time and the subsequent 45 ns as production time. Output for positions and velocities was recorded at every 1000 steps.

## Results

The dielectric properties of aqueous electrolyte solutions as dielectric inside in a parallel nanoslit of graphene hold significant importance for our understanding of the hydration and complexation behavior of ions. This understanding is crucial for comprehending related fundamental mechanisms in liquids, such as electric conductivity, structure saturation, dielectric friction, and kinetic depolarization. The study of these properties allows us to explore how ions interact with water molecules, providing insights into the underlying processes that govern ionic behavior in solution.

**Figure 1:**
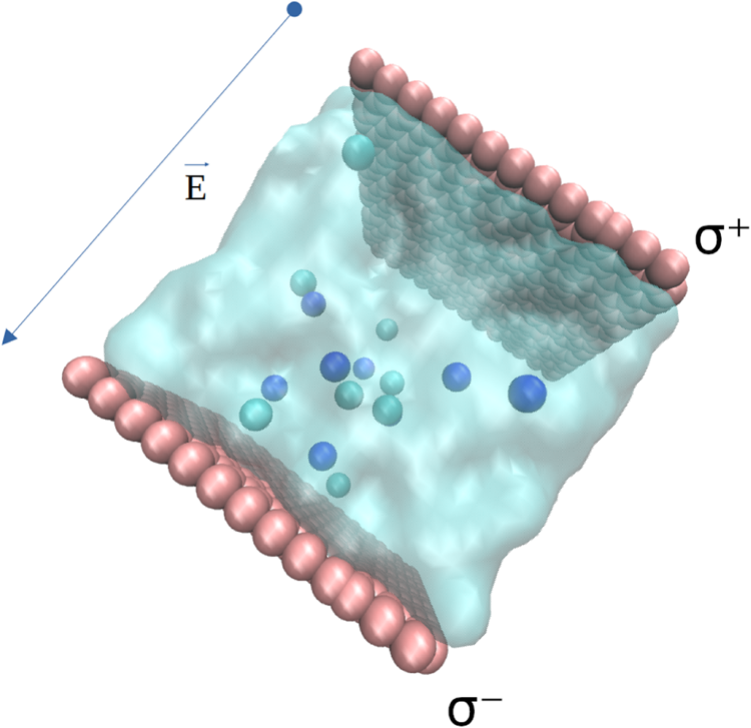
Image of the 0.5 molal NaCl solution in water, situated between two graphene nanoslits with zero surface charge (zero volts).

In Figure 1, we can see the image of a 0.5 molal NaCl solution in water at zero volts, where the graphene nanoslits can have a surface charge due to the individual charge of each carbon atom. Each carbon atom has an identical charge value, either positive or negative, depending on its location. This is illustrated in the figure, with surface density of charges described as σ^+^ and σ^–^ respectively.

**Figure 2:**
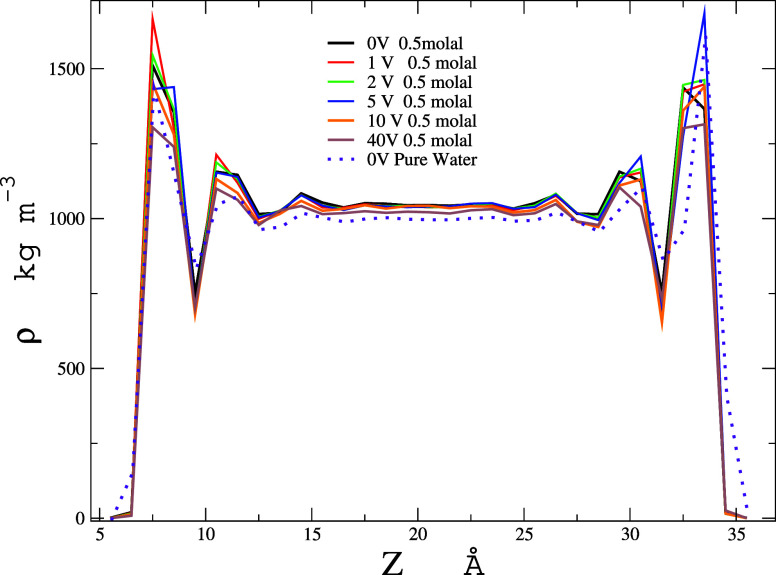
Density profile along the z -direction under varying electrical potentials between the plates for 0.5 molal initial solution of NaCl and water.

In the Figure 2, we present the density profile along the *Z*-axis of the simulation cell, where significant increases in density can be observed near the graphene. This is a distinctive feature of confined water, characterized by its high density in the vicinity of the graphene nanoslit. Our results demonstrate that the pronounced density layering of water on the graphene surface dissipates within approximately 9 Å (three times the diameter of a water molecule), consistent with previous studies of water on flat solid surfaces. This observation not only aligns with prior research but also underscores the unique behavior of water in confined spaces, revealing important insights into the interactions at the nanoscale.^39−41^

**Figure 3:**
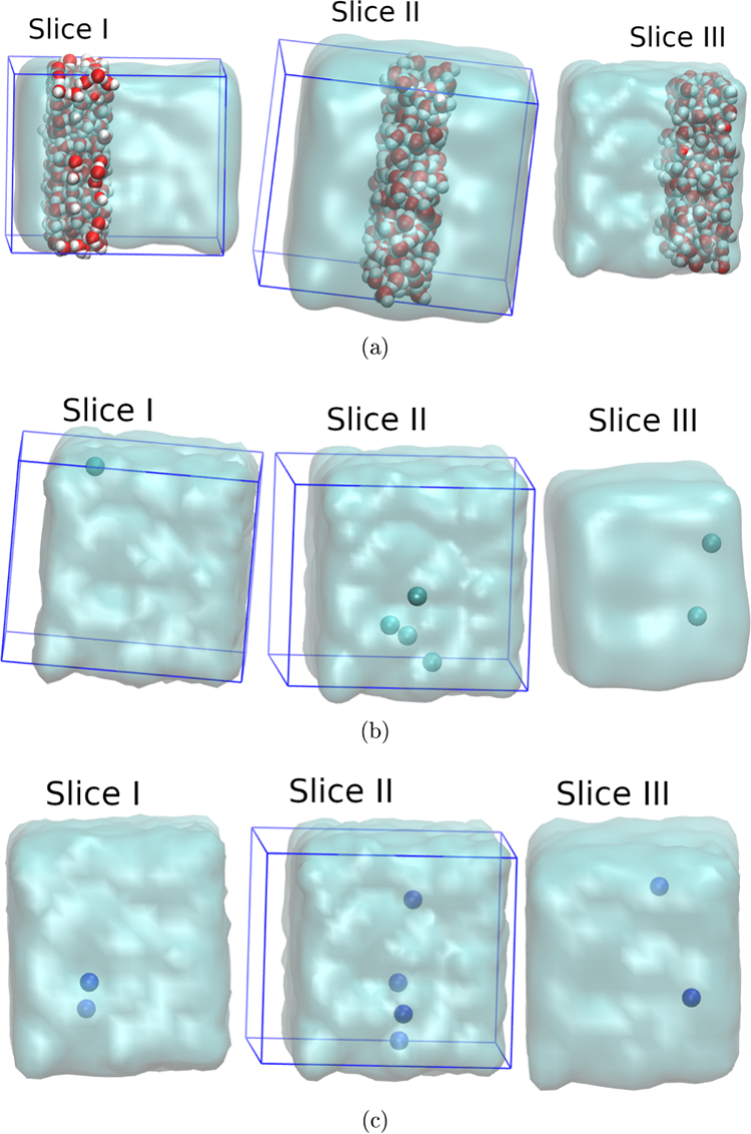
Regions I, II, and III, into which the simulation cell is divided, form the basis of our comprehensive analysis of thermodynamic, structural, and dielectric properties. These figures correspond to 0 V.

In the bulk region within the limits of 15 and 25 Å along the *Z*-axis, the density is 1039 kg m^–3^ until 10 V, which corresponds to the experimental value of the 0.98 molal solution, since the bulk contains 240 water molecules and an average of 8 pairs of ions, as described in the Figure 3 and to be analyzed later. This observation is in excellent agreement with experimental data^42^ and highlights the accuracy of our simulation in capturing the behavior of the solution at the nanoscale. At 40 V, we observed a decrease in bulk density to 1020 kg m^–3^, which is attributed to the presence of 4 ion pairs in 246 water molecules. This density value corresponds to a 0.45 molal experimental bulk solution, which is consistent with expectations. The molality calculation is performed using the following eq 5.
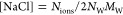
5

The consistency with the experimental value further validates our model and provides confidence in the reliability of the simulated density profile for confined water. The dashed violet line represents the calculation of pure water without ions at 0 V, where the bulk density is also 994 kg m^–3^, a value accurately reproduced by the FBA/ϵ model. This concordance underscores the model’s ability to replicate the density of pure water under these specific conditions with an error of less than 0.5%,^18^ reinforcing the robustness and reliability of the simulation framework. The alignment with established experimental data further emphasizes the validity of our approach in modeling the density profiles of confined water systems.

Once we have the simulations, the analysis is conducted by dividing the simulation cell into distinct zones, as depicted in the Figure 3. This illustration clearly shows the molecules at zero volts and the different zones into which the cell was divided for this study. Thus, we have Region I, which is confined within 0.55 nm < Z < 1.65 nm along the *Z*-axis. Region II is defined by the values 1.65 nm < Z < 2.45 nm along the *Z*-axis, and Region III is delineated by the range 2.45 nm < Z < 3.55 nm along the *Z*-axis. This segmentation allows for a detailed examination of each region’s unique characteristics, providing deeper insights into the behavior of confined water. By systematically analyzing these distinct areas, we can more accurately capture the nuances of water’s interaction with the graphene nanoslit, enhancing our understanding of its complex properties at the nanoscale.

**Figure 4:**
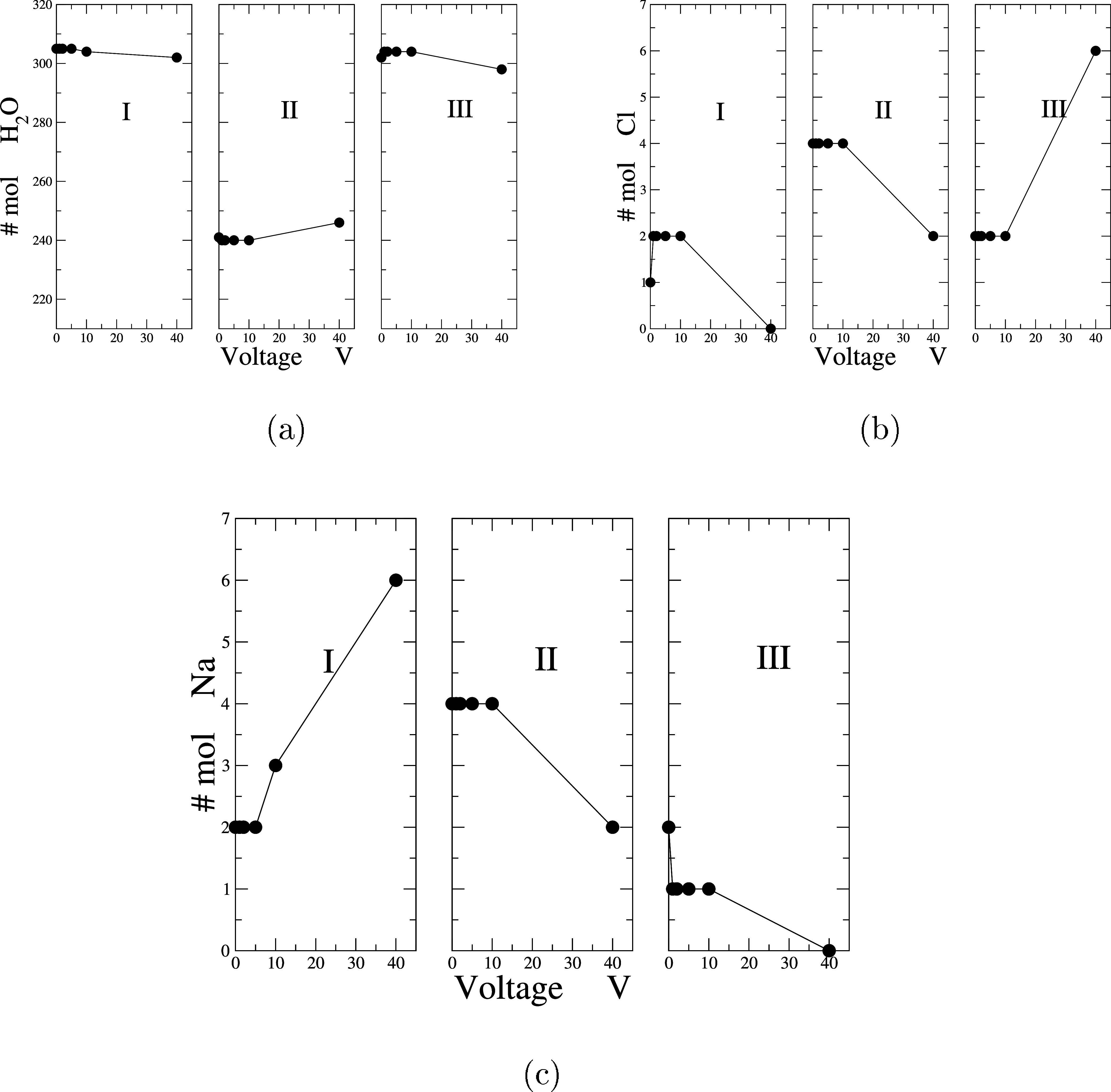
(a) Representation of the simulation cell in three regions. In each region, the number of H2O molecules versus the voltage is presented. (b) Representation of the simulation cell in three regions. In each region, the number of Cl molecules versus the voltage is presented. (c) Representation of the simulation cell in three regions. In each region, the number of Na molecules versus the voltage is presented.

In Figure 4, it is evident that the number of H_2_O molecules in Regions I and III increases, leading to a corresponding rise in density in these zones, as depicted in Figure 2. In contrast, Region II, the bulk solution contains 240 water molecules and an average of 4 pairs of ions, resulting in a 0.9559 molal solution until 10 V, however, at 40 V, it increases in the bulk while decreasing at the interfaces.

Understanding whether this number of molecules is sufficient to accurately reproduce thermodynamic and dielectric properties is crucial. According to a recent study,^43^ with a cutoff radius of 0.9 nm and starting from 256 molecules of water, properties such as density, enthalpy of vaporization (Δ*H*), and dielectric constant are well reproduced. Therefore, the values reported in this work are well-supported by these findings.

Regarding Cl^–^ ions, it is observed that the number of molecules increases monotonically with voltage and is predominantly found in Region II, without migrating to the cathode, despite being negatively charged up to 10 V, at 40 V there is a decrease near the anode and in the bulk, leading to an accumulation of chloride ions near the cathode. For Na^+^ ions, there is a noticeable attraction toward the anode, primarily in Region I. Although the number of Na^+^ ions is small, they are drawn to this region, leaving Region III with an excess of Cl^–^ ions. It is observed that from 10 V onward, there are more sodium ions near the anode. This detailed analysis underscores the importance of region-specific behavior in understanding the complex interactions within the confined water system.

### Dielectric Constant ϵ_⊥_

The alterations in water’s unique dipole moment and the restricted flexibility of the molecule, significantly influence its behavior as a dielectric medium. The static dielectric constant, ϵ, of a polar liquid is connected to the thermal equilibrium fluctuations of polarization in the absence of an external field.^44^ Since polarization fluctuations are extensive and vary with the dielectric body’s shape, confinement notably impacts these long-range interactions.

The static dielectric constant of a liquid can be determined from MD trajectories using various methods. A common approach for calculating the dielectric constant of a bulk liquid involves analyzing fluctuations in the total dipole moment using the methodology outlined by Neumann,^45^ utilizing eq 6 for the total dipole moment.
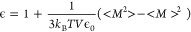
6

where *k*_B_ is the Boltzmann constant, *T* is the absolute temperature, ϵ_0_ is the vacuum permittivity and *V* represents the volume.

However, in confined systems, it has been experimentally observed that the component perpendicular to the nanoslit contributes the most to the value of the dielectric constant.^24,25^ This observation has also been reported in various theoretical studies.^26−27,28,29,30^,^32^ Based on these works, we calculate ϵ_⊥_ here, following the procedure outlined below.

**Figure 5:**
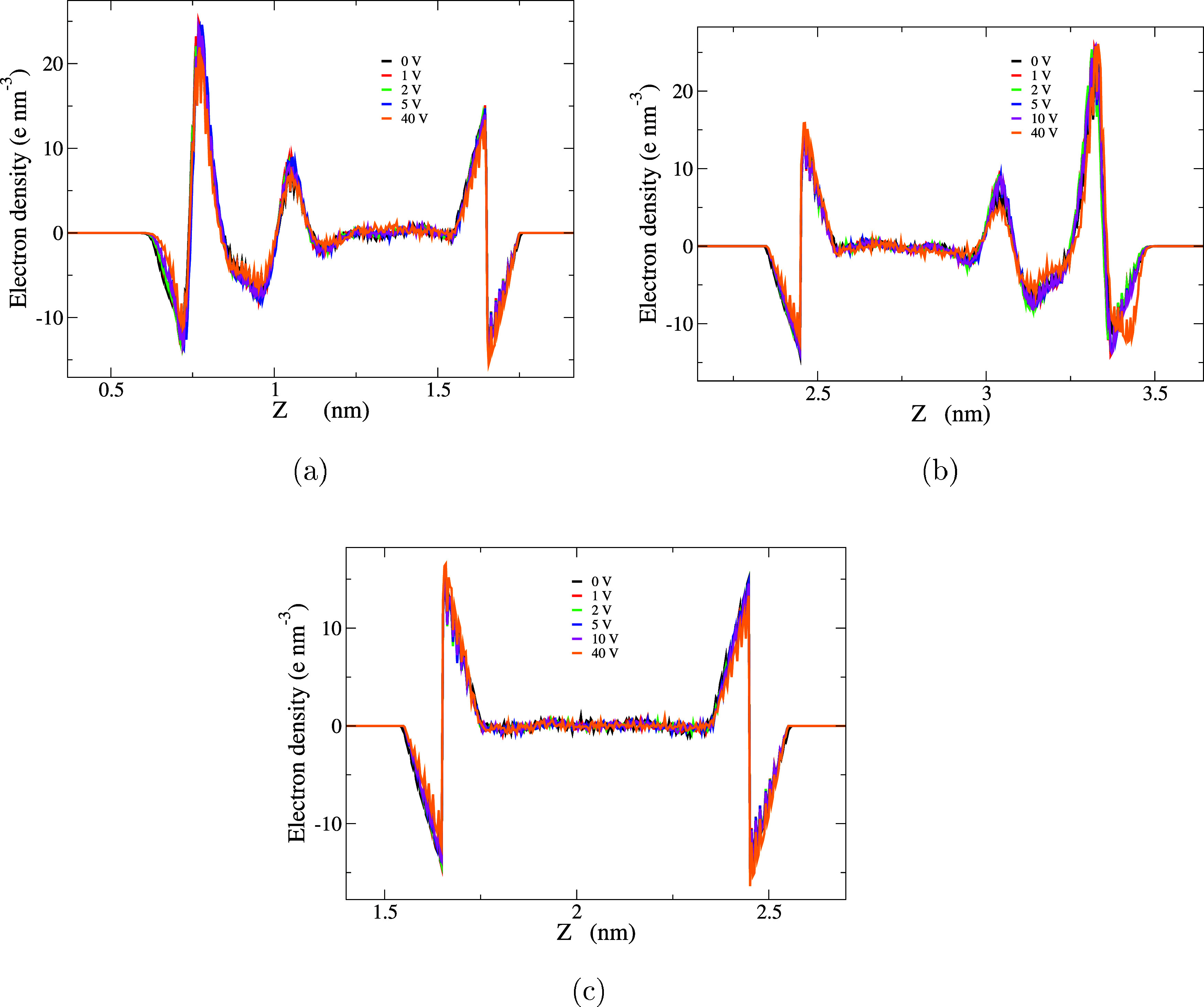
Spatial distribution of the charge density ρ_*q*_ for the 0.5 molal concentration of NACl and water with the models FBA/ε for water and NACl/ε for the ions, at different electrical potential (a) ρ_*q*_ for region I (b) ρ_*q*_ for region III and (c)ρ_*q*_ for region II.

To compute the dielectric constant under confinement, we utilized the approach outlined by Ballenegger et al.^31^ This method starts with determining the charge density profile, as described in eq 7. The profile is obtained by sampling data every 1000 time steps, up to 50 ns of the simulation. The resulting profiles are illustrated in Figure 5 for the confined 0.5 molal solution.
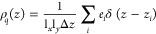
7

Subsequently, we determined the normal polarization density *m*_⊥_ averaged laterally across each region marked in Figure 3, as expressed in eq 8.
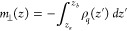
8where *z*_*a*_ corresponds to the lower limit and *z*_*b*_ to the upper limit of each region.

Thus, to obtain the total polarization, we perform the following integral
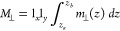
9where l*_x_* and l*_y_* represent the lateral area of the channel. The normal component of the polarization correlation function is subsequently computed as
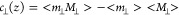
10

Thus, to calculate the dielectric constant ε_⊥_, we perform the following calculation
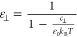
11

**Figure 6:**
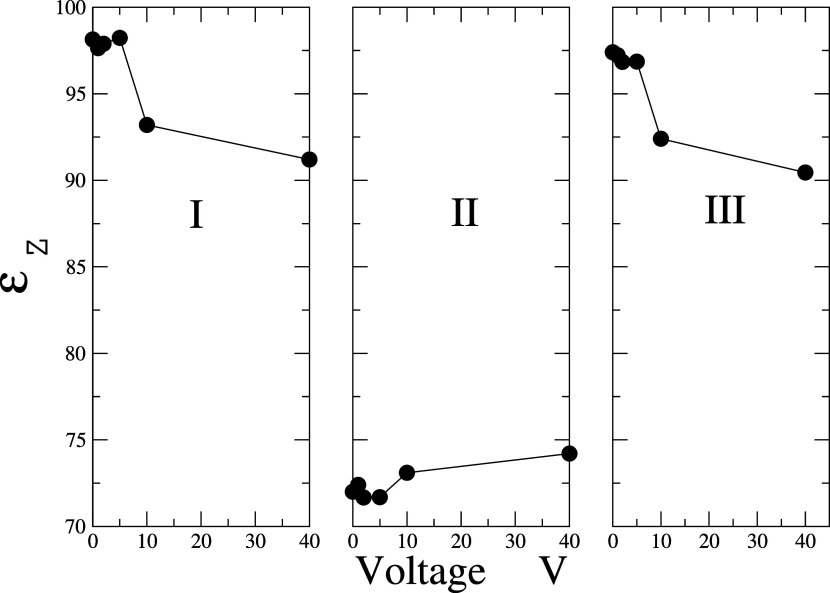
Dielectric constant ε_⊥_ at different electric potentials. The SPC/ε rigid model is represented by the red squares and the FBA/ε model by the black circles.

The values obtained for the dielectric constant ε_⊥_ are shown in Figure 6, where it can be seen that in the experimental 0.5 molal concentration.

In the bulk, outside of confinement and under standard conditions, the dielectric constant of a 0.5 molal solution of water and NaCl is isotropic, with a value of ϵ_bulk_ = 73. For the FAB/ϵ and NaCl/ϵ, the bulk dielectric constant is ϵ_bulk_ = 71. These values were calculated in this work.

As illustrated in Figure 6, the dielectric constant in regions I and III decreases as the electric potential difference increases, and in the bulk it increases. This is due to the presence of ions in each region, since there is a different average number as the electric potential increases.

Since the ions are monovalent, their individual dipole moment is zero; however, when in contact with water, they create distinct collective dipole moments. In this manner, in contact with water, they become hydrated, which alters the dielectric constant of the water and results in a well-defined coordination number, which is accurately reproduced by the models used in this work.

The dielectric constant depends on the temporal variation of the dipole moment. Hence, by calculating the instantaneous dipole changes of the molecules in the region of interest, the dielectric constant can be determined by averaging over time. This method correlates the interaction with neighboring molecules in the calculation.

### Finite System Kirkwood *g*-Factor, *G*_*k*_

Given that the orientations of molecules are crucial to dielectric properties, we have assessed the polarization factor *G*_*k*_. This factor measures the equilibrium fluctuations of the systemś collective dipole moment and is linked to the orientational correlation function. Kirkwood theorized that it is possible to express ϵ in terms of a short-range orientational correlation function.^46^ Accordingly, we evaluate the polarization factor *G*_k_ in this context.,^47^
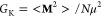
12Here, *N* is the total number of molecules, and *M* is the sum total of dipoles μ in the system. Local orientational correlations are averaged out by thermal motion after the first few coordination shells.

**Figure 7:**
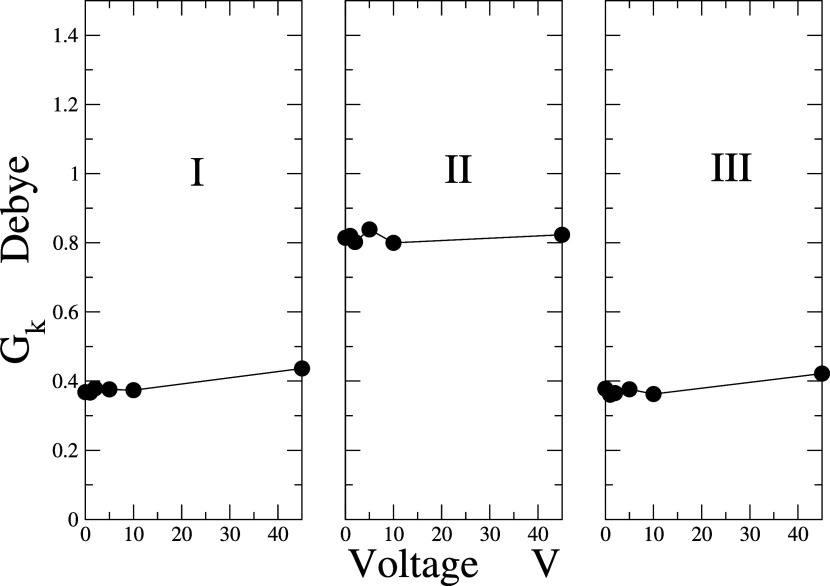
The finite system Kirkwood *g*-factor, *G*_k_ of water molecules concerning the differential electrical potential in each region of the cell.

The polarization values G_*k*_ within the pore, as shown in Figure 7, reveal values less than 1 in all three regions. This suggests that the molecule is less polarized compared to its initial state, indicating a reduction in the asymmetry of the charge distribution. It is also evident that the polarization in regions I and III is lower than in region II, which indicates that in this area, the molecules experience a notable reduction in the asymmetry of the charge distribution.

### Capacitance, C_⊥_

The slit-pore configuration of two charged plates can also be regarded as a capacitor on a molecular scale. In fact, a more detailed analysis can be conducted to examine the behavior of the dielectric constant in each system. Since the dielectric constant varies within the pore, it is expected that the capacitance between the plates will also differ. For two parallel plates, the perpendicular capacitance with a dielectric is calculated using the following expression.
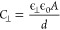
13

**Figure 8:**
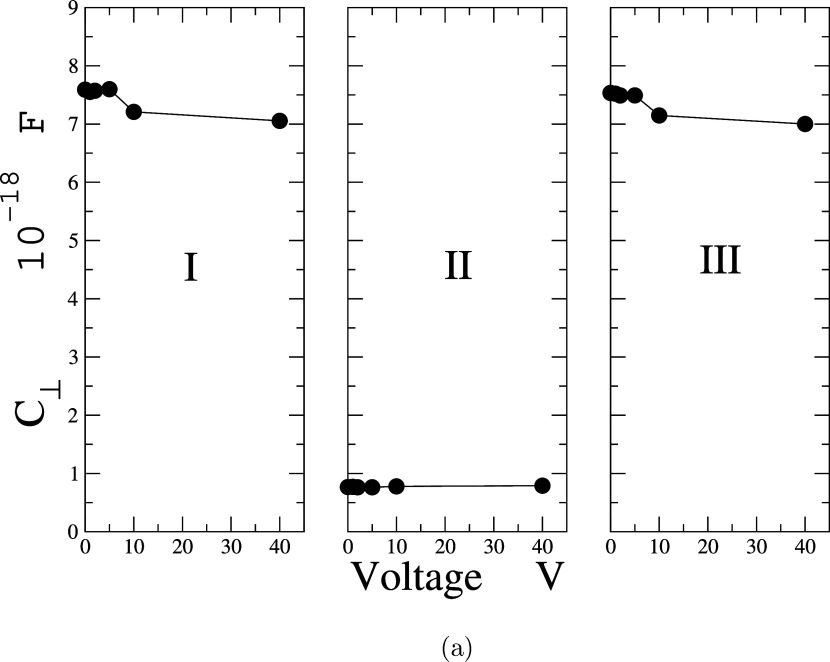
Capacitance vs voltage in each region of the system.

Here, ϵ_⊥_ is the perpendicular dielectric constant of the dielectric, ϵ_0_ is the dielectric constant of the vacuum, *d* is the effective thickness of each region of the double layer, and *A* is the *X*,*Y* area. By using the average perpendicular dielectric constant inside the pore, the results for the perpendicular capacitance for different electric potentials are shown in Figure 8.

## Conclusions

Molecular Dynamics simulations were carried out to explore the behavior of a 0.5 molal NaCl solution in water when confined between graphene nanoslit plates, with varying electric fields applied by change the charge density. These generate a electrical potential difference across the plates, simulating a nanocapacitor environment. For these simulations, the flexible FAB/ϵ model and the NaCl/ϵ model were employed, both of which are well-suited for capturing the intricate interactions between ions, water molecules.

As the electric potential increases, notable changes in the bulk phase are observed, primarily driven by the concentration and mobility of the ions in the solution. In the bulk region, an increase in density occurs as the ions respond to the field. However, within a critical range of potential difference, up to approximately 10 V, no significant structural or dynamic changes are evident, suggesting that the system maintains a quasi-stable state within this range. Beyond this threshold, as the potential difference is further increased, the system undergoes a more pronounced transformation, with ions actively migrating toward the anode and cathode. This migration induces the charging of the nanoslit plates, enhancing the system’s overall electrostatic potential and contributing to further alterations in ion distribution and molecular orientation within the confined space.

The dielectric constant, which is a key parameter in determining the system’s response to the electric field, is predominantly influenced by the components perpendicular to the field. However, the system’s behavior is further complicated by the polarization effects, as described by the G_*k*_ parameter and changes in the dipole moment. These effects are particularly evident in the direction parallel to the electric field, where significant changes in molecular orientation and charge distribution are observed. This indicates that the perpendicular component of the dielectric constant response plays a crucial role in modulating the overall confinement process, influencing both the structural properties of the confined solution and the dynamic behavior of the ions within the nanoslit.
